# Dataset of groundnut plant leaf images for classification and detection

**DOI:** 10.1016/j.dib.2023.109185

**Published:** 2023-04-28

**Authors:** Aishwarya M．P．, A. Padmanabha Reddy

**Affiliations:** Vijayanagara Sri Krishnadevaraya University, Ballari-583101, Karnataka, India

**Keywords:** Classification of leaf diseases, Image dataset, Diagnosis of disease, Computer Vision

## Abstract

The use of machine learning is rapidly expanding across many industries, including agriculture and the IT sector. However, data is essential for machine learning models, and a substantial amount of data is required prior to training a model. We have collected data of groundnut plant leaves in the form of digital photographs taken in the Koppal (Karnataka, India) area with the assistance of a pathologist in natural settings. Images of leaves are categorized into six distinct groups according to their condition. Collected images are pre-processed and the processed images of groundnut leaves are kept in 6 folders as: the “healthy leaves” folder with 1871 images, the “early leaf spot” folder with 1731 images, the “late leaf spot” folder with 1896 images, the “Nutrition deficiency” folder with 1665 images, the “rust” folder with 1724 images, and the “early rust” folder with 1474 images. The total number of images in the dataset is 10361. This dataset will be useful to train and validate deep learning and machine learning algorithms for groundnut leaf disease classification and recognition. Disease detection in plants is crucial for limiting crop losses and our dataset will help disease detection in groundnut plants. This dataset is freely accessible to public at https://data.mendeley.com/datasets/22p2vcbxfk/3 and at https://doi.org/10.17632/22p2vcbxfk.3

**Specifications Table**

**Table:** 

Subject	Computer Science, Agricultural Science.
Specific subject area	Image processing
Type of data	Raw data consisting of image of the size 1200 × 800 pixels having RGB color in JPG format.
How the data were acquired	Data acquisition was done using canon mark II D camera sensor size of APS-C, dimension of 122.4 × 92.6 × 69.8m Horizontal and vertical resolution pf 72dpi,bit depth 24,monitor 3.0 inch,autofocus of and maximum continuous shooting rate 5fs. Data annotation was done manually and Data acquisition was done with the help of a pathologist.
Data format	JPG Format, Raw Data
Description of data collection	Images are collected in rainy and sunny days. A total of 3059 raw images are collected and only interested areas are cropped using cropping tool and created a dataset consist of six classes with 10361 images using augmentation technique with image size of 256 × 256 pixels.
Data source location	The following two groundnut farm fields of Karnataka are used for data collection: 1. Chilwadigi village groundnut farm field in Koppal district (Latitude: 15.39783 and Longitude: 76.161604). 2. Yelburga Taluk groundnut farm field in Koppal district (Latitude: 15.618803 and Longitude: 76.027645).
Data accessibility	Repository name: Mendeley Data [6] Data identification number: 10.17632/22p2vcbxfk.3 Direct URL to data: https://data.mendeley.com/datasets/22p2vcbxfk/3

## Value of the Data

• Machine learning and deep learning models are tested using data.
• For data analytics to be effective, substantial amounts of relevant data are required.
• To train a computer to recognize photographs with human precision, a huge amount of data must be tagged accurately.
• The image dataset of groundnut leaves assists in the diagnosis of plant diseases and contributes to the development of smart agriculture.

## 1 Objective

Implementing machine learning models for disease detection in plants using images of their leaves is an exciting area for future study and development. In order to create efficient machine learning systems, researchers in this area require easy access to representative datasets. However, real-world groundnut datasets for identifying plant diseases are rare [1], [2]. Our review of the relevant literature shows that there is currently no standard image dataset of groundnut leaves in terms of size and images, etc. Therefore, we believe it is critical to create and disseminate such a dataset to promote study of plant disease detection using machine learning [3]. In the opinion of many leading experts in the field of machine learning, the benefits of this field have not yet been fully exploited for societal goods like healthcare and agriculture. Our goal is to create a standardized agricultural dataset that will help spread the word about machine learning and its potential applications for the general public in some way, no matter how small.

## 2 Data Description

All of the images are from the Koppal area, as shown in Table 1. During the Kharif and Rabi seasons, the data is gathered. There are six distinct groups represented in this data set [6]:  early leaf spot, late leaf spot, rust, early symptoms of rust (early rust), healthy leaf and nutrition deficiency. We see a breakdown of early leaf spot and late leaf spot according to when groundnuts are planted and harvested. Early leaf spot appears in the first month of cultivation, while late leaf spot appears in the vicinity of three months. Each disease is described in detail below:

**Table 1:** The conditions of leaf along with the number of images for each condition.

**Category**	**Number of images**
Healthy leaf	1871
Early leaf spot	1731
Late leaf spot	1896
Nutrition Deficiency	1665
Rust	1724
Early Rust	1474
**Total**	**10361**

### 2.1 Early Leaf Spot

**Fig. 1:**
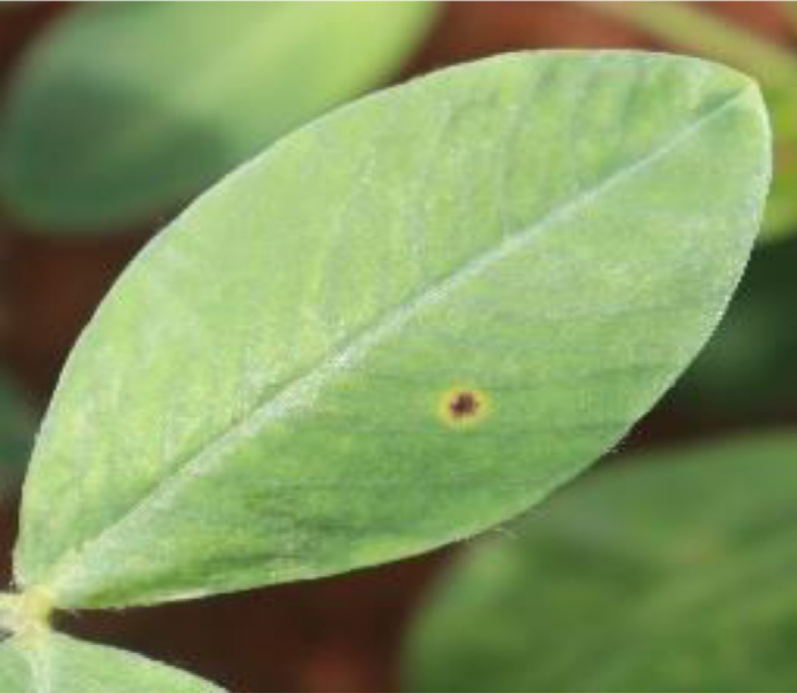
Early leaf spot.

As its name suggests, *cercospora arachidicola*, the fungus responsible for early leaf spot, manifests itself early on in the crop's development after planting. Lesions on the underside of a leaf are typically a lighter shade of brown, and they are surrounded by a yellow halo [3]. During wet seasons, disease tends to spread rapidly. A digital camera is used to take pictures of the damaged leaves. Early leaf spot was captured in one of the 1731 images taken, as shown in Fig. 1.

### 2.2 Late Leaf Spot

**Fig. 2:**
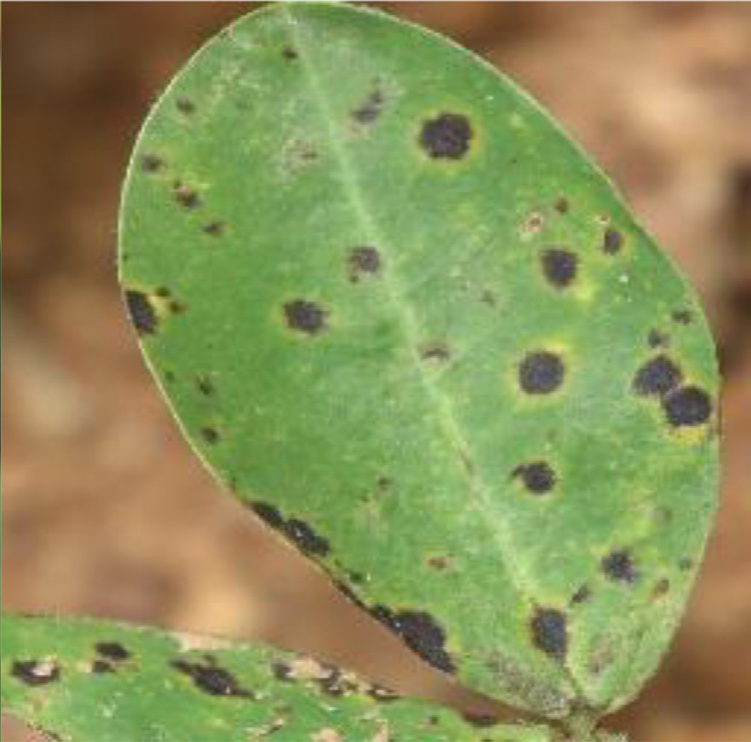
Late leaf spot.

*Cercospora personata*, the causal agent of late leaf spot, was widely seen in all of the mature farmer fields. Around 7–8 weeks after planting, infections begin. Lesions on leaves look like small, circular, dark brown spots without a yellow halo, and their undersides and upper side are a carbon black color [3]. For further analysis, a digital camera captures a total of 1896 images. For an example of a late leaf spot, see Fig. 2.

### 2.3 Nutrition Deficiency

**Fig. 3:**
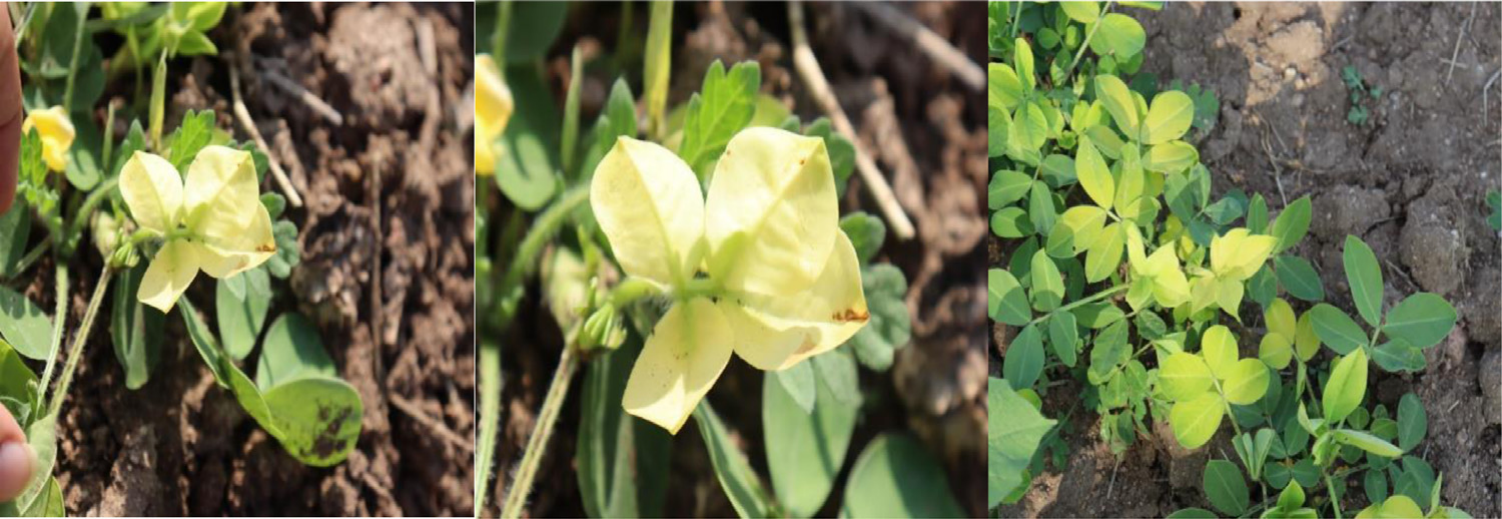
Nutrition deficiency.

Lack of Sulphur and Iron contributes significantly to nutritional deficiencies. When Sulphur is lacking, yellowing starts in the middle leaves and works its way up the plant, but when nitrogen is lacking, it starts in the lower leaves and works its way up to the younger leaves. Iron deficiency shows up first on the leaves of young plants. A lack of chlorophyll causes the leaves to turn a sickly white color. Totaling 1665 images, all were taken with a digital camera. Leaf discoloration due to a lack of nutrients is depicted in Fig. 3.

### 2.4 Rust

**Fig. 4:**
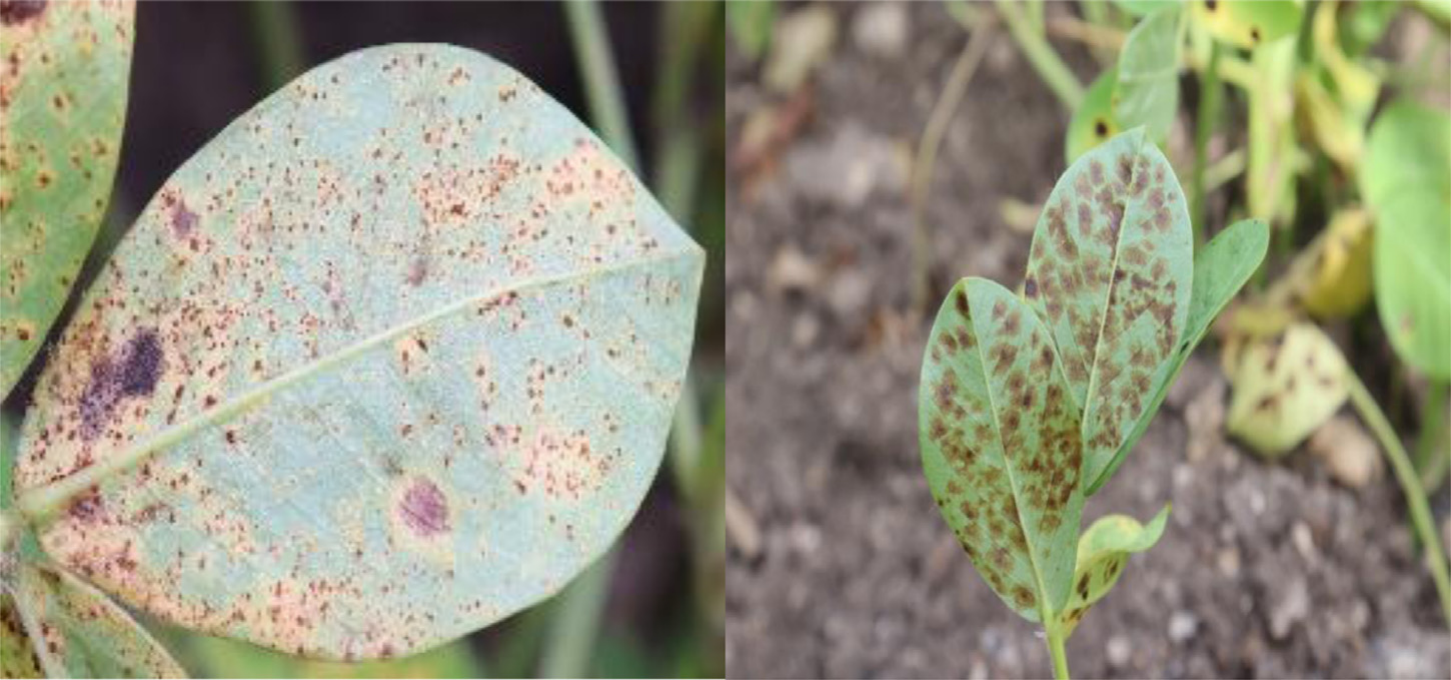
Rust.

Peanut rust disease is a major biotic stress factor because it reduces economic productivity by lowering pod and fodder yields and degrading oil quality. The fungus *Puccinia arachidis* is responsible for this disease. The fungus parasite that causes rust needs living plants in order to reproduce. Rust diseases tend to flourish in humid, mild climates. Rust is transmitted from diseased to healthy plants via spores. Diseases like rust can quickly spread after being watered, as the spores can be carried by the wind or the water. Infections can only spread on wet surfaces. A total of 3198 digital photographs are taken including early symptoms of rust and severity of rust in groundnut plant as shown in Fig. 4. A picture of rust is shown in Fig. 4.

## 3 Experimental Design, Materials and Methods

### 3.1 Data Acquisition

The images were captured from the groundnut farm field using a high resolution digital camera of 18mm-55mm. The affected leaves images are captured with the help of pathologist who helped in identifying a diseases using observation method [5]. In total five types of diseased images were collected namely early leaf spot, late leaf spot, nutrition deficiency, rust, early symptoms of rust.

### 3.2 Data Pre-Processing

#### 3.2.1 Cropping and Resizing

Those working in deep learning and machine learning can benefit from using a crop tool to isolate the target region of a leaf image. Most deep learning model architecture requires uniformity in input image size, so after cropping, images are resized to 256 × 256.

#### 3.2.2 Data Augmentation

**Fig. 5:**
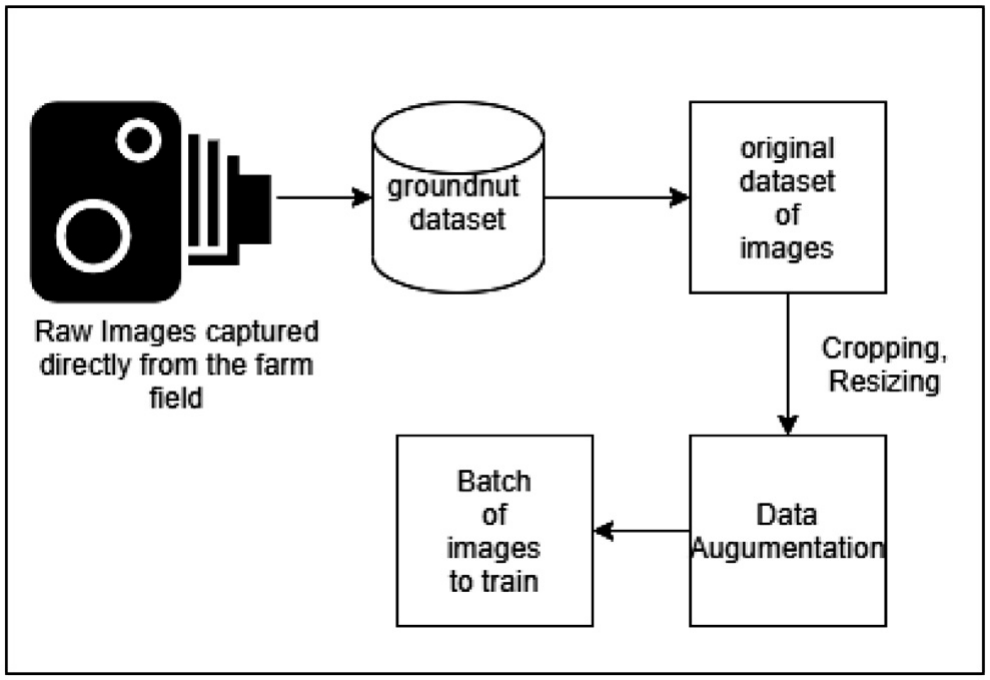
Data expansion on the group of image.

Processing is done to the images that were taken using digital camera out in the field. The data augmentation approach is useful for boosting the amount of data by adding duplicates of the data that have been minimally modified from their original state [4] as shown in Fig. 5. As part of the augmentation process, the photos were flipped horizontally, shear range and zooming were both set to 0.2, and the images were rotated at an angle of 360 degrees.

### 3.3 Specifics of the Implementation

#### 3.3.1 Densenet

**Fig. 6:**
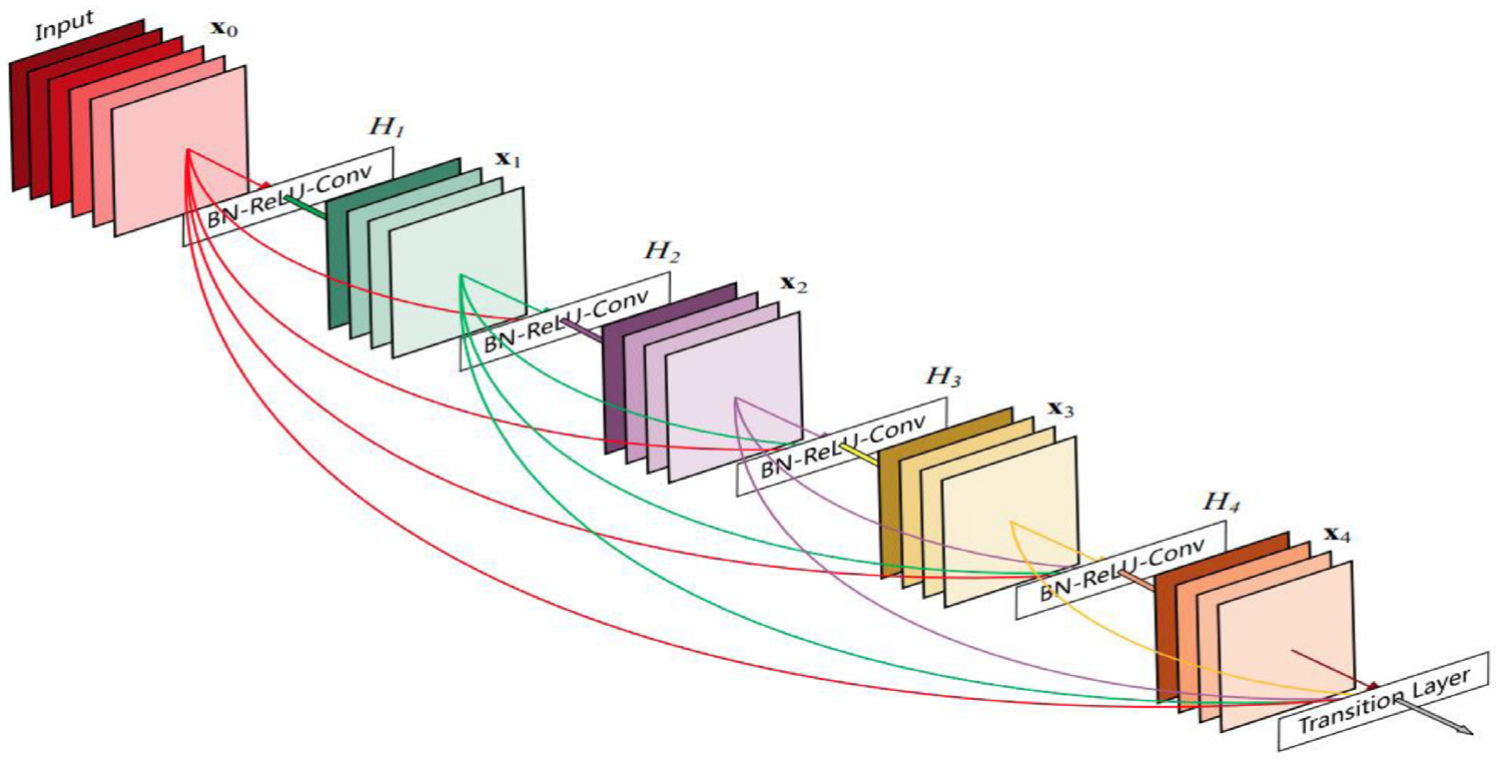
Densenet-169 Architecture.

Densenet-169 is one of the architectures in the Densenet family. It has 169 layers and is a popular choice for deep learning classification applications due to its layered structure. When compared to the other Densenet architectures that have fewer layers, it has a significantly smaller number of trainable parameters. We can classify Densenet-169 and the other Densenet frameworks as a family of very reliable deep learning architectures due to their ability to avoid the issue of vanishing gradients have an efficient feature propagation tactics, reduce the number of trainable parameters, and promote the reuse of features. Fig. 6 presents the layered architecture of the Densenet-169, which was utilized in this particular research endeavour.

Convolutional layers, dense layers, maxpool layers (also known as fully connected layers), and transition layers are all components of the design. The ReLU activation function is used consistently throughout the model's design, whereas the SoftMax activation is reserved for the very last layer. The image's features are extracted by the convolutional layers, while the inputs' dimensionality is decreased by the maxpool layers. The flatten layer is followed by the fully connected layers, which function as an artificially generated neural network and get their input from a single array provided by the flatten layer. Table 2 illustrates the specifics of the layered architecture in its entirety.

**Table 2:** Model summary.

conv5_block31_1_conv (Conv2D)	None, 6, 6, 128)	204800
conv5_block31_1_bn (Batch Normalization)	(None, 6, 6, 128)	512
conv5_block31_1_relu (Activation)	(None, 6, 6, 128)	0
conv5_block31_2_conv (Conv2D)	(None, 6, 6, 32)	36864
conv5_block31_concat (Concatenate)	(None, 6, 6, 1632)	0
conv5_block32_0_bn (Batch Normalization)	(None, 6, 6, 1632)	6528
conv5_block32_0_relu (Activation)	(None, 6, 6, 1632)	0
conv5_block32_1_conv (Conv2D)	(None, 6, 6, 128)	208896
conv5_block32_1_bn (Batch Normalization)	(None, 6, 6, 128)	512
conv5_block32_1_relu (Activation)	(None, 6, 6, 128)	0
conv5_block32_2_conv (Conv2D)	(None, 6, 6, 32)	36864
conv5_block32_concat (Concatenate)	(None, 6, 6, 1664)	0
bn(Batch Normalization)	(None, 6, 6, 1664)	6656
relu (Activation)	(None, 6, 6, 1664)	0
conv2d (Conv2D)	(None, 4, 4, 1024)	15336448
max_pooling2d (MaxPooling2D)	(None, 1, 1, 1024)	0
flatten (Flatten)	(None, 1024)	0
dense (Dense)	(None, 256)	262400
dropout (Dropout)	(None, 256)	0
dense_1 (Dense)	(None, 128)	32896
dropout_1 (Dropout)	(None, 128)	0
dense_2 (Dense)	(None, 5)	645
Total params: 28,275,269		
Trainable params: 28,116,869		
Non-trainable params: 158,400		

### 3.4 Experimental Results

The proposed groundnut leaf dataset is used to train and test the densenet model's performance in identifying and categorizing the disease; the dataset consist of 10361 photographs labelled with 6 classes. For the purposes of validating groundnut leaf diseases, only photographs of infected leaves are taken from the dataset. Images from the train set are used to train a model, which is then used to classify images from the test set. The densenet-169 model, with RMSprop acting as optimizer, has accomplished an overall validation accuracy of 99.83%.

#### 3.4.1 Evaluation Metrics

In order to derive the model for making predictions, we looked at the following four matrices they are accuracy, precision, recall and F1-score.

• The phrase true positive is used to describe the proportion of positive results that are accurately detected (TP).
• False negatives occur when predictions turn out to be wrong (FN).
• If the model erroneously classifies an outcome as positive, we call that a false positive (FP).
• When the model accurately predicts the negative class, we say that result is a true negative (TN).
  (1) $$\text{Accuracy}=\frac{TP+TN}{TP+FP+TN+FN}$$
  (2) $$\text{Precision}=\frac{TP}{TP+FP}$$
  (3) $$\text{Recall}=\frac{TP}{TP+TN}$$
  (4) $$F1-\text{Score}=2\;\times \frac{Precision\;x\;Recall}{Precision+Recall}$$

#### 3.4.2 Result Analysis

**Fig. 7:**
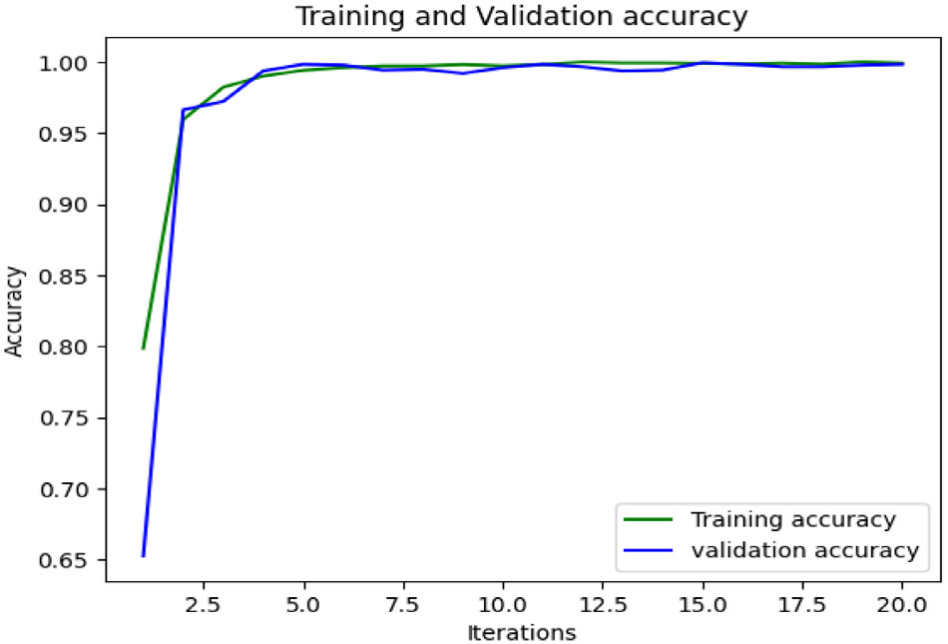
Model accuracy and validation accuracy of densenet-169 classifier.

**Fig. 8:**
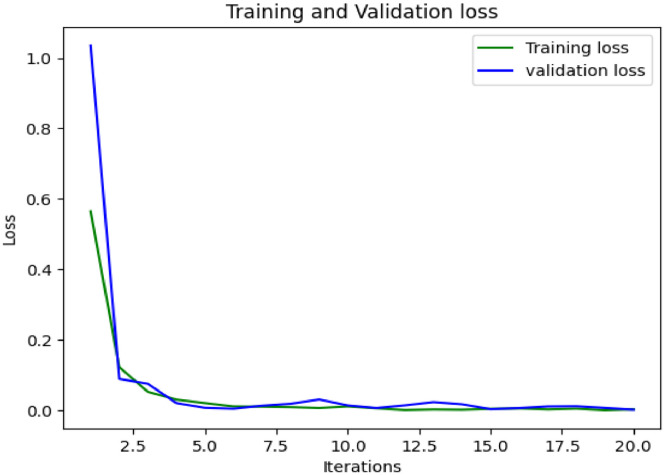
Model loss and validation loss of densenet-169 classifier.

The densenet-169 model's accuracy, recall, and F1-score are listed in Table 3. For late leaf spot, precise forecasts can be made. Fig. 7 displays the accuracy graphs in relation to the number of iterations, and it also includes a comparison of the training accuracy and the validation accuracy in relation to the number of iterations. Fig. 8 presents the loss in model and validation.

**Table 3:** Densenet-169 classifier classification report for the proposed dataset.

	Precision	Recall	F1-score
Late leaf spot	1.0000	1.0000	1.0000
Early Rust	0.9966	1.0000	0.9983
Early Leaf Spot	0.9943	1.0000	0.9971
Rust	1.0000	0.9942	0.9971
Nutrition Deficiency	1.0000	0.9972	0.9986
Accuracy			0.9983

**Fig. 9:**
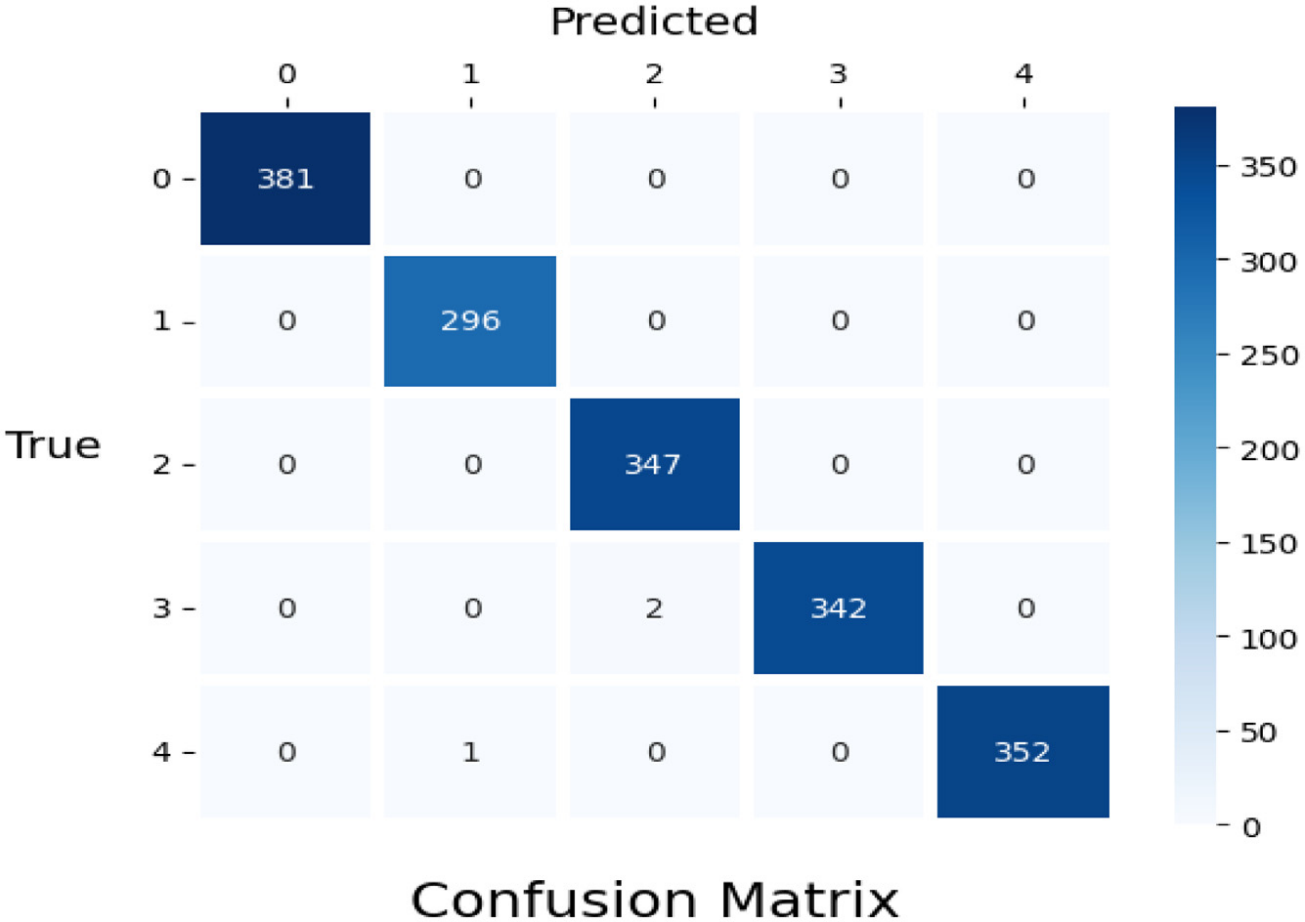
Confusion matrix for densenet-169 classifier.

As we can see, Fig. 9 confusion matrix reveals that the model is little unclear about the difference between nutrition deficiency leaf and rust leaf. Whereas two photos of late leaf spot were misidentified as rust, and one image of rust was misidentified as early rust. On the other hand, the model correctly categorizes the other classes.

## 4 Conclusion

Research into deep learning methods is proceeding at a rapid pace. Based on the results of the Densenet framework (Densenet-169), the Densenet is capable of accurately detecting and classifying infected leaves of the groundnut plant of the proposed dataset. The model's success in training and testing on the groundnut leaf dataset demonstrates the dataset's worth. Densenet model produced a 99.83% success rate and accuracy values of the suggested model are found to be greater. The article achieves its purpose by creating a standardized agricultural data that will contribute to increasing awareness of machine learning as well as its potential uses.

## Ethics Statements

1. This article is an original work by the author that has not been published elsewhere.
2. The article is not presently under consideration for publication anywhere else.
3. The article reflects the authors own research and analysis in a truthful and complete manner.

We agree with the above statements and declare that this submission follows the policies of Solid State Ionics as outlined in the Guide for Authors and in the Ethical Statement.

## CRediT authorship contribution statement

**Aishwarya M．P．:** Methodology. **A. Padmanabha Reddy:** Supervision.

## Declaration of Competing Interest

The authors declare that they have no known competing financial interests or personal relationships that could have appeared to influence the work reported in this paper.

## Data Availability

Dataset of groundnut plant leaf images for classification and detection (Original data) (Mendeley Data).
